# The haustorium as a driving force for speciation in thallus-forming *Laboulbeniomycetes*

**DOI:** 10.1186/s43008-021-00087-7

**Published:** 2022-01-31

**Authors:** Danny Haelewaters, Maarten Lubbers, André De Kesel

**Affiliations:** 1 Faculty of Science, University of South Bohemia, Branisovska 31, 350 07 České Budějovice, Czech Republic; 2 Research Group Mycology, Department of Biology, Ghent University, K.L. Ledeganckstraat 35, 9000 Ghent, Belgium; 3 Harvard University Herbaria, 22 Divinity Avenue, Cambridge, MA 02138 USA; 4 Institute of Biology Leiden, Leiden University, Sylviusweg 72, 2333 BE Leiden, The Netherlands; 5 Meise Botanic Garden, Nieuwelaan 38, 1860 Meise, Belgium

**Keywords:** Ectoparasitic fungi, Haustorium, *Herpomyces*, Host specificity, Integrative taxonomy, *Laboulbeniales*, One-Host-One-Parasite

## Abstract

*Laboulbeniomycetes* is a class of fungi that have obligate associations with arthropod hosts, either for dispersal (order *Pyxidiophorales*) or as biotrophic parasites (orders *Herpomycetales* and *Laboulbeniales*). Here, we focus on *Herpomycetales* and *Laboulbeniales*, which include fungi that form thalli, 3-dimensional, multicellular units of 1000 s of cells. Based on recently published data regarding patterns of speciation, we present the One-Host-One-Parasite model (1H1P) for haustorial thallus-forming *Laboulbeniomycetes*. We hypothesize that taxa with haustoria, rhizoidal structures that make contact with the host’s body cavity, have very strict host specificity. For taxa without haustoria, the microhabitat—as selected by the host—governs host shifting, presence or absence of the fungus, abundance, effective host range, and geographic distribution. We make suggestions for future research including fluorescent labeling of waxy lipids and mass spectrometry. These techniques have the potential to generate the data necessary to evaluate the here proposed 1H1P hypothesis for *Herpomycetales* and *Laboulbeniales*.

## THALLUS-FORMING *LABOULBENIOMYCETES*

Fungi in the class *Laboulbeniomycetes* are obligately associated with arthropods—either for dispersal (order *Pyxidiophorales*) or as biotrophs (orders *Herpomycetales* and *Laboulbeniales*). Species of *Pyxidiophora* are characterized by a complex, three-morph life-cycle; they have one sexual morph and two independent asexual morphs (Lundqvist 1980; Blackwell et al. 1986a, 1986b; Blackwell and Malloch 1989; Kirschner 2003; Jacobs et al. 2005). Taxa in *Herpomycetales* and *Laboulbeniales* differ from other fungi in their non-hyphal multicellular units of up to several thousand cells, or thalli, which are directly formed from two-celled ascospores (Thaxter 1896; Benjamin 1971; Tavares 1985; Haelewaters et al. 2019b). These thalli are characterized by determinate growth. No asexual morphs are known in these orders. Species descriptions in thallus-forming *Laboulbeniomycetes* have been traditionally based on morphological features, but recent work has shown that the morphological species concept (sensu de Queiroz 1998, 2007) does not always hold to the phylogenetic (or unified) species concept (Haelewaters et al. 2018, 2019c; Haelewaters and Pfister 2019).

## PATTERNS OF SPECIFICITY

*Laboulbeniales* and *Herpomycetales* are unique for displaying host, habitat, and position specificity. The majority of taxa are host-specific; they occur on a certain host genus or even species (Richards and Smith 1954; De Kesel 1996a, b). However, when one species occurs on phylogenetically disparate hosts, these hosts are sympatric; they invariably occur in the same microhabitat. For example, *Stichomyces conosomatis*, which is specific to species of the genus *Sepedophilus* (*Coleoptera*, *Staphylinidae*), has been found on *Speonemadus algarvensis* (*Coleoptera*, *Leiodidae*) in subterranean caves where specimens of both beetle genera co-occur (Reboleira et al. 2017). During a study of ant nest inquilines—arthropods that live in ant nests and have an obligatory symbiotic relationship with the ants, *Rickia wasmannii*, until then considered strictly host-specific to ants of the genus *Myrmica* (*Hymenoptera*, *Formicidae*), was found infecting phylogenetically far-distant arthropods, mites, and a fly larva (Pfliegler et al. 2016).

Species of *Laboulbeniales* and *Herpomyces* produce sticky ascospores that are primarily transmitted through direct contact (De Kesel 1996a, b)—such as mating, grooming behavior of social insects, and intra- and inter-generational contacts in dense (overwintering) aggregations. The success of ascospores adhering to an integument and developing into a mature thallus not only depends on the characteristics of the integument but also the habitat conditions selected by the host. De Kesel (1996a, b), rearing insects on different soils in the laboratory, was able to successfully grow *Laboulbenia slackensis* on atypical hosts in the same conditions as preferred by its typical host, *Pogonus chalceus*. This discovery has pushed research on the ecology of *Laboulbeniales* and habitat specificity in particular, because under natural conditions *L. slackensis* is only found associated with a few strictly halobiont species of *Pogonus*, *Pogonistes*, and *Syrdenus* (*Coleoptera, Carabidae*) (Santamaría 1998). However, one host species, *Cafius xantholoma* (*Coleoptera, Staphylinidae*), which shares the coastal, wet, and saline environment of *P. chalceus*, is host to *Laboulbenia littoralis*. *Laboulbenia littoralis* and *L. slackensis*, are morphologically very similar, and we assume that the fungus has shifted from one host to the other as a result of host sympatry (habitat specificity), followed by divergent natural selection and reproductive isolation (De Kesel and Haelewaters 2014).

Another type of specificity goes to the extreme; certain taxa of *Herpomycetales* and *Laboulbeniales* are only found on a particular position of the host’s integument; this is referred to as position specificity. For example, *Herpomyces periplanetae* occurs on the antennae of *Periplaneta* cockroaches (*Blattodea*, *Blattidae*), *Chitonomyces unciger* is only ever observed on the left posterior claw of male diving beetles of the genus *Laccophilus* (*Coleoptera*, *Dytiscidae*), and *Laboulbenia hyalopoda* is found exclusively on the last abdominal segment of *Paradromius linearis* (*Coleoptera, Carabidae*) (De Kesel 1998; Goldmann and Weir 2012; Wang et al. 2016). Hosts with position-specific taxa very often carry one or more other species elsewhere on their integument (Goldmann and Weir 2012). Without data from molecular phylogenetic studies, morphology-based conclusions about this type of specificity remain problematic; are all of these taxa biological species?

## SPECIES AND MORPHOTYPES

Authors have followed two opposing views when describing forms based on morphology alone. One view recognizes species that are strictly position specific and/or only occur on a given sex of the host (= sex-of-host specificity). Benjamin and Shanor (1952) described six species of *Laboulbenia* on *Bembidion grapii* (*Coleoptera, Carabidae*), each restricted to a given position on the host. The alternative view recognizes forms that are related to host, host sex, and position on the integument, as morphotypes of given biological species. However, without molecular data and data from transmission studies (including host specificity and host shifting), it is impossible to draw limits among (1) morphologically similar thalli that have different host species, (2) morphologically different thalli on different sexes of the same host species, or (3) morphologically different thalli that occupy different positions on the same host specimen (Scheloske 1969, 1976). This is why we have advocated an integrative approach to the taxonomy of thallus-forming *Laboulbeniomycetes* (Haelewaters et al. 2018, 2019a, c)—an effort that has long been adopted in many other groups of fungi (Wijayawardene 2019), including *Aspergillus* (Pringle et al. 2005), *Cortinarius* (Stefani et al. 2014), *Geastrum* (Accioly et al. 2019), *Helvella* (Skrede et al. 2017), *Leptographium* (Yin et al. 2019), *Octospora* (Sochorová et al. 2019), *Ophiocordyceps* (Araújo et al. 2018), *Phialocephala* (Grünig et al. 2008), *Protoparmelia* (Singh et al. 2015), and *Tranzscheliella* (Li et al. 2017). This push towards an integrative fungal taxonomy has been met with some resistance among laboulbeniologists faced with the impracticability of performing molecular work, who perhaps perceive it as a threat to the long-standing traditional morphology-based species descriptions. We do, however, want to emphasize the importance of alpha taxonomy (Haelewaters et al. 2021a), as generating sequences for *Laboulbeniales* remains challenging, especially for specimens from dried entomological collections (Weir and Blackwell 2001; Haelewaters et al. 2015; Sundberg et al. 2018a). Besides, many researchers lack access to sufficient funding or equipment to generate molecular data. These researchers are often based in tropical areas where most of the world’s undescribed species are still to be found (Hawksworth and Rossman 1997; Haelewaters et al. 2021b). Because tens of thousands of *Herpomycetales* and *Laboulbeniales* taxa are still to be discovered (Weir and Hammond 1997), we advise taxonomists to continue working with available resources and techniques with the understanding that future molecular phylogenetic work may confirm or shift species limits by supporting or rejecting the taxonomic value of used morphological characters and their variability. We believe collaboration between fungal molecular systematists and classically trained taxonomists should be the end goal.

## ONE-HOST-ONE-PARASITE, OR THE HAUSTORIUM THEORY

As we explore generalized speciation patterns of taxa in *Herpomycetales* and *Laboulbeniales* using combined morphological, molecular, and ecological data from individual thalli, we can possibly link some of these patterns to morphological or life history traits. One such candidate trait is the haustorium. This rhizoidal structure penetrates the host’s integument to make contact with the haemocoel, possibly to provide additional holdfast and increase surface area for uptake of nutrients and water. Haustoria can be simple or branched, and single (in *Laboulbeniales*) or multiple (in *Herpomycetales*) per thallus. All *Herpomyces* species form haustoria. Benjamin (1971) thought that all *Laboulbeniales* formed haustoria, but recent work (Tragust et al. 2016; Reboleira et al. 2021) found no evidence for penetration in five species of ant- and millipede-associated *Laboulbeniales*. In fact, compared to the superficially attached *Laboulbeniales*, haustorial *Laboulbeniales* are rare and represented in only 13 of 146 described genera (Thaxter 1931; Tragust et al. 2016): *Arthrorhynchus*, *Coreomyces*, *Dimeromyces*, *Gloeandromyces*, *Hesperomyces*, *Hydrophilomyces*, *Laboulbenia*, *Microsomyces*, *Moschomyces*, *Rhizomyces*, *Stigmatomyces*, *Trenomyces*, and *Thaumasiomyces*. Note that in a given genus, only some species may produce haustoria, as is the case in *Gloeandromyces*, *Laboulbenia*, and *Stigmatomyces*. Accumulating evidence for the presence of non-haustorial taxa in *Laboulbeniales* challenges the idea of the group being ectoparasitic as a whole; *Laboulbeniales* may instead occupy several positions on the symbiosis spectrum ranging from ectobiont (that is, they are externally attached) commensals to ectoparasites. One paper even presented evidence for a mutualistic role of *Laboulbeniales* for their hosts, in protecting them from infection by entomopathogenic fungi (Konrad et al. 2015).

**Fig. 1:**
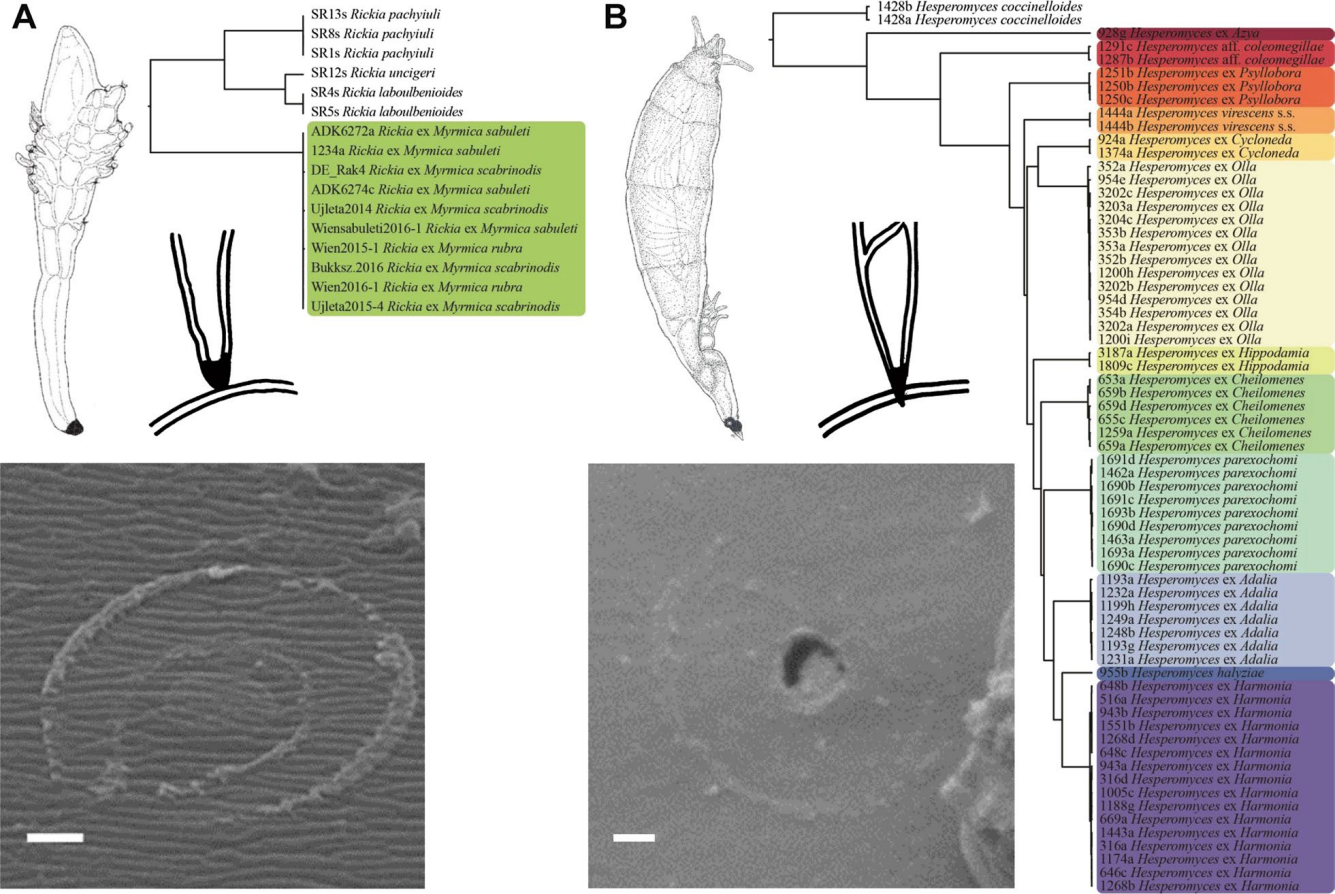
Visualization of the proposed One-Host-One-Parasite model. **A**. *Rickia wasmannii*, a single phylogenetic species with *Myrmica* hosts in phylogenetically unrelated species groups; no penetration through the host’s integument into the haemocoel; and a horseshoe-shaped imprint around a circular inner ring at the otherwise unaffected host integument (scanning electron micrograph, from J. Billen, X. Espadaler, A. Tartally, and S. Tragust). **B**. *Hesperomyces virescens*, a complex of multiple species, each species corresponding to a phylogenetic clade with isolates from thalli removed from a given host; penetration through the integument; and a circular 1-µm diameter penetration pore (scanning electron micrograph, from M. Lubbers). Bars = 1 µm. Color schemes for monophyletic clades from https://colorbrewer2.org by C.A. Brewer, Geography, Pennsylvania State University

We consider that, due to the invasive nature of their haustoria, *Herpomycetales* and haustorial *Laboulbeniales* maintain close interactions with their hosts, possibly involving adaptations to the hosts’ defense systems and leading to escape-and-radiate coevolution (Ehrlich and Raven 1964). This kind of coevolution involves a process of stepwise adaptation and counter-adaptation; a host develops a new defense mechanism, to “escape” association with a given parasite and diversify. The given parasite can evolve new counter mechanisms, ultimately resulting in physiological adaptation. Enhanced by their exclusively sexual mode of reproduction, these developments lead to an evolutionary arms race, involving specialization and increasing reproductive isolation. This is analogous with Dobzhansky’s (1946) idea emphasizing physiological aspects in *Drosophila* speciation. For example, *Hesperomyces virescens* forms a haustorium and is in fact a complex of many near-cryptic species, each with their own host (Haelewaters et al. 2018). In contrast, *Rickia wasmannii* does not form a haustorium and is a single phylogenetic species with different *Myrmica* hosts that are placed in phylogenetically unrelated species groups (Haelewaters et al. 2019a). Both examples provide support for the haustorium theory (Fig. 1).

We propose the One-Host-One-Parasite (1H1P) model, with *Hesperomyces* as prime example (Haelewaters and De Kesel 2020). We hypothesize that the presence or absence of haustoria determines speciation in thallus-forming *Laboulbeniomycetes*. In the presence of a haustorium or haustoria, host specificity is likely high or more strict. Host recognition mechanisms, which are necessary for spore germination and subsequent penetration of the integument, may be affected or even blocked when ascospores land on a host other than the main host. In contrast, in the absence of haustoria, there are no such developmental barriers and transmitted ascospores can develop on different and even unusual host taxa given these co-occur in a particular microhabitat. Xenotransmission is thought rare in nature because of strict habitat choices of hosts, but we can force development of thalli on unusual hosts under the right conditions in the laboratory (De Kesel 1996a, b) and there are a few examples from the field (De Kesel and Haelewaters 2014; Pfliegler et al. 2016; Reboleira et al. 2017). For non-haustorial *Laboulbeniales* the potential host range is probably wider than the natural host range; occasional hosts (Nebenwirten) and accidental hosts (Zufallswirten) have been recognized for certain taxa of *Laboulbeniales*. These are the result of fortuitous encounters when the main host species has overlapping niches with other arthropods occurring in the same microhabitat (Scheloske 1969). Since ascospores are not airborne (Huldén 1983) nor long-lived (De Kesel 1996a, b), direct transmission by contact is a key factor here. For example, some species of *Clivina* (family *Carabidae*, subfamily *Scaritinae*) serve as accidental hosts for *Laboulbenia anoplogenii* after encountering the parasite’s typical hosts, which are beetles in subfamilies *Harpalinae* and *Pterostichinae* (Santamaría 1998). The fungus may not persist on these alternative hosts, but accidental transmission probably has played an important role in speciation processes of *Laboulbeniales* (Rossi 2011; De Kesel and Haelewaters 2014). Since successful development of a fungus population on its natural host is determined by the habitat (De Kesel 1996a, b), radiation of non-haustorial *Laboulbeniales* is entirely governed by the habitat choice of any host on which ascospores land.

We suggest that losing the need for an haustorium, created opportunities for *Laboulbeniales* to shift more easily towards hosts occupying the same or a similar, suitable habitat. This change allowed them to radiate into megadiverse host groups such as *Carabidae* and *Staphylinidae*. Abandoning the haustorium has widened the parasite’s host range and provided opportunities for radiation, probably forcing the evolution of alternative nutrient uptake mechanisms.

Early on in the studies of *Laboulbeniales*, Cavara (1899) proposed that thalli might receive nutrients and water from the environment by absorption through their (sterile) appendages. Since many species of *Laboulbeniales* lack appendages, this claim had long been rejected, until results from experimental work supported Cavara’s suggestion (De Kesel 1997). Since we now know that at least some species of *Laboulbeniales* definitely have no haustorium (Tragust et al. 2016; Reboleira et al. 2021), alternative explanations regarding *Laboulbeniales* nutrition are needed. We suggest that non-haustorial *Laboulbeniales* take up waxy lipids as nutrients produced by the host (Tavares 1985; Stanley and Nelson 1993). Such lipid transfers have been shown in the arbuscular mycorrhizal *Glomeromycota*, which take up fatty acids containing lipids from plant roots (Keymer and Gutjhar 2018).

## FUTURE RESEARCH

Experimental work should be directed towards investigating nutrient uptake mechanisms in *Laboulbeniales*. Three alternative methods could be employed:

(1) The use of fluorescence-labeled waxy lipids to monitor waxy lipid uptake into the non-haustorial *Laboulbeniales* cells. As it has only been hypothesized that non-haustorial *Laboulbeniales* take up lipids from the arthropod integument, it remains uncertain which type(s) of lipids this could be. A selection of waxy lipids could therefore be fluorescently labeled with BODIPY (Wang et al. 2018). BODIPY has already been used for various pathogenic organisms such as the pathogenic rice fungus *Magnaporthe oryzae* (Wang et al. 2018) and the parasitic worm *Schistosoma mansoni* (Furlong et al. 1995). Living insect specimens with thalli of *Laboulbenia* would need to be collected in the field and bred under laboratory conditions. The site of infection, around the point of contact, should be treated with fluorescently labeled waxy lipids. Fluorescent signals in the thallus cells can then be observed using confocal fluorescence microscopy.

(2) Non-labeled waxy lipids could be investigated in cells of *Laboulbeniales* with a lipidomics approach (Wenk 2006). Using techniques such as mass spectrometry, various lipids can be detected and characterized. Both host as well as *Laboulbeniales* cells should be profiled for waxy lipids. If there is a significant similarity between these profiles, this could indicate waxy lipid uptake by the fungus.

(3) The use of stable isotope labeling to monitor lipid uptake by *Laboulbeniales* cells. Previous experiments with protozoan parasites have shown that ^13^C-labeled precursors (glucose, amino acids, fatty acids) can be taken up by host cells (Kloehn et al. 2016). Whether these labeled metabolites are taken up by the parasite can be detected through liquid chromatography–mass spectrometry (LC/MS), allowing researchers to identify active parasite pathways in vivo (Kloehn et al. 2016). Applying this methodology to *Laboulbeniales*, we suggest feeding arthropod hosts on a diet of ^13^C-labeled precursors. If the basal-most receptacle cells (especially the foot cell) of *Laboulbeniales* show presence of ^13^C-labeled molecules with LC/MS detection, this could prove the uptake of specific molecules by the fungus. One should prevent spillage of ^13^C labeled food by the beetle (via grooming via the mouth parts), otherwise the thallus may directly get in contact with this food and take up ^13^C directly via its highly absorbing appendages.

Previous research has shown that limited transmission is possible in (haustorial) *Hesperomyces virescens* between different host species (Cottrell and Riddick 2012). One could suggest this contradicts our 1H1P hypothesis. However, interspecific transmission has only been observed for *C. septempunctata*, while not for the three other host species tested (Cottrell and Riddick 2012). We believe habitat specificity may allow for transmission, as the hosts were bred in a micro-habitat for an extended time. If separated, these interspecific transmissions could either be ecological dead-ends or result in reproductive isolation over time. This should be tested in long-term multigenerational experiments, with *Hesperomyces virescens* as the model organism.

## CONCLUDING REMARKS

Recent work with thallus-forming *Laboulbeniomycetes* has shown that the field is faced with multiple challenges. These range from understanding patterns of speciation to unexplored territories such as exploring the function of the sterile appendages of non-haustorial *Laboulbeniales*. If one thing has become increasingly clear, it is that morphology alone is not sufficient to draw accurate species limits, due to the existence of near-cryptic species (Crous et al. 2021) and species of which the morphology is affected by the position they occupy on the host integument (Haelewaters and Pfister 2019; Sundberg et al. 2021). Rather, an integrative approach, combining data from multiple sources, is necessary in fungal taxonomy (Cao et al. 2021; Maharachchikumbura et al. 2021), more specifically the taxonomy of *Herpomycetales* and *Laboulbeniales* (e.g., Goldmann and Weir 2012; Goldmann et al. 2013; Haelewaters et al. 2018, 2019a, b, c; Sundberg et al. 2018b, 2021; Haelewaters and Pfister 2019; Gutierrez et al. 2020). For this reason, we propose to accompany descriptions of new taxa at all ranks with independent lines of evidence from morphology, DNA, and ecology (host associations). This is in line with the most recent best-practice advocated by the *International Commission on the Taxonomy of Fungi* (ICTF) on how to describe new fungal species (Aime et al. 2021).

In aiming to understand how speciation in thallus-forming *Laboulbeniomycetes* is mediated, we have hypothesized here that the presence or absence of haustoria is a determining factor. In the presence of a haustorium or multiple haustoria, this automatically leads to the 1H1P model. As for non-haustorial *Laboulbeniales*, there is no doubt that thallus development depends on both host and habitat. Whereas opportunities for host shifting and radiation increase when different host species share the same microhabitat, successful transmission is contingent upon the nature of the ascospores (not airborne, short-lived; Huldén 1983; De Kesel 1996a, b). The ascospores of all *Laboulbeniomycetes* are uniform throughout the group—always single-septate (although the position of the septum may differ) and built to stick. This guarantees that ascospore transmission between infected and uninfected hosts is highly promoted by direct, mostly intra-specific contact. Studies in specificity and host shifting of *Laboulbeniales* should also focus on various aspects of transmission, an important bottleneck, especially in situations where hosts are forced to move among habitats because of climate change and human influences (Carlson et al. 2020). Changes in the distribution range of the hosts may affect transmission patterns. To fully understand transmission, we cannot at this point exclude infections to occur through asexual or free-living stages. To be able to detect these stages of *Laboulbeniales* in environmental next-generation sequencing data, we recommend laboulbeniologists to generate curated sequences of *Laboulbeniales* and make them public. Only recently, a clade containing both genera known only as asexual morphs (*Chantransiopsis* and *Tetrameronycha*) and ones only known as sexual morphs (*Subbaromyces*) was revealed in the class based on molecular phylogenetic analyses (Goldmann and Weir 2018; Blackwell et al. 2020). By increased sampling of fresh material and generating new sequences, it would be no surprise that many more major discoveries will be made in coming years—hopefully shedding more light to this group of enigmatic fungi.

## Data Availability

Not applicable.

## Abbreviations

1H1P: One-Host-One-Parasite model
^13^C: Carbon-13
BODIPY: Boron dipyrromethene
ICTF: International Commission on the Taxonomy of Fungi
LC/MS: Liquid chromatography–mass spectrometry
